# A pragmatic and scalable strategy using mobile technology to promote sustained lifestyle changes to prevent type 2 diabetes in India and the UK: a randomised controlled trial

**DOI:** 10.1007/s00125-019-05061-y

**Published:** 2020-01-09

**Authors:** Arun Nanditha, Hazel Thomson, Priscilla Susairaj, Weerachai Srivanichakorn, Nick Oliver, Ian F. Godsland, Azeem Majeed, Ara Darzi, Krishnamoorthy Satheesh, Mary Simon, Arun Raghavan, Ramachandran Vinitha, Chamukuttan Snehalatha, Kate Westgate, Soren Brage, Stephen J. Sharp, Nicholas J. Wareham, Desmond G. Johnston, Ambady Ramachandran

**Affiliations:** 1 India Diabetes Research Foundation, Chennai, India; 2 Dr. A. Ramachandran’s Diabetes Hospitals, 110, Anna Salai, Guindy, Chennai, 600 032 India; 3 Department of Metabolism, Digestion and Reproduction, Faculty of Medicine, Imperial College, London, SW7 2AZ UK; 4 Department of Medicine, Siriraj Hospital, Mahidol University, Bangkok, Thailand; 5 School of Public Health, Faculty of Medicine, Imperial College, London, UK; 6 Department of Surgery and Cancer, Faculty of Medicine, Imperial College, London, UK; 7 MRC Epidemiology Unit, University of Cambridge School of Clinical Medicine, Box 285 Institute of Metabolic Science, Cambridge Biomedical Campus, Cambridge, CB2 0QQ UK

**Keywords:** Behavioural change, Diabetes prevention, Glycosylated haemoglobin A_1c_, Lifestyle modification, Mobile technology, Prediabetes, Screening, Short message service

## Abstract

**Aims/hypothesis:**

This randomised controlled trial was performed in India and the UK in people with prediabetes to study whether mobile phone short message service (SMS) text messages can be used to motivate and educate people to follow lifestyle modifications, to prevent type 2 diabetes.

**Methods:**

The study was performed in people with prediabetes (*n* = 2062; control: *n* = 1031; intervention: *n* = 1031) defined by HbA_1c_ ≥42 and ≤47 mmol/mol (≥6.0% and ≤6.4%). Participants were recruited from public and private sector organisations in India (men and women aged 35–55 years) and by the National Health Service (NHS) Health Checks programme in the UK (aged 40–74 years without pre-existing diabetes, cardiovascular disease or kidney disease). Allocation to the study groups was performed using a computer-generated sequence (1:1) in India and by stratified randomisation in permuted blocks in the UK. Investigators in both countries remained blinded throughout the study period. All participants received advice on a healthy lifestyle at baseline. The intervention group in addition received supportive text messages using mobile phone SMS messages 2–3 times per week. Participants were assessed at baseline and at 6, 12 and 24 months. The primary outcome was conversion to type 2 diabetes and secondary outcomes included anthropometry, biochemistry, dietary and physical activity changes, blood pressure and quality of life.

**Results:**

At the 2 year follow-up (*n* = 2062; control: *n* = 1031; intervention: *n* = 1031), in the intention-to-treat population the HR for development of type 2 diabetes calculated using a discrete-time proportional hazards model was 0.89 (95% CI 0.74, 1.07; *p* = 0.22). There were no significant differences in the secondary outcomes.

**Conclusions/interpretation:**

This trial in two countries with varied ethnic and cultural backgrounds showed no significant reduction in the progression to diabetes in 2 years by lifestyle modification using SMS messaging.

**Trial registration:**

The primary study was registered on www.ClinicalTrials.gov (India, NCT01570946; UK, NCT01795833).

**Funding:**

The study was funded jointly by the Indian Council for Medical Research and the UK Medical Research Council.

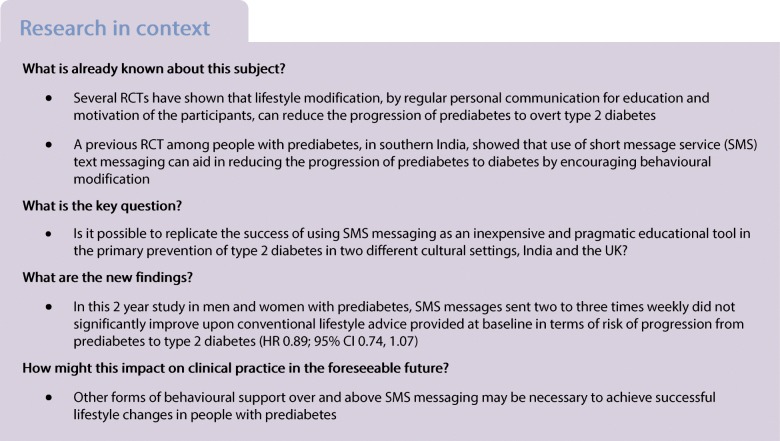



## Introduction

The public health challenge of type 2 diabetes is set to worsen as the prevalence rises from 425 million people globally in 2017 to 629 million by 2045 [1]. Diabetes is preceded by a period of intermediate hyperglycaemia (prediabetes), during which lifestyle interventions have been shown to reduce progression to diabetes in several RCTs [2–4].

The interventions in the initial diabetes prevention RCTs were labour intensive and difficult to scale up to reach large numbers of people at risk. Simple and scalable approaches to educate and motivate at-risk individuals to make behavioural changes using mobile phone short message service (SMS) text messages have been developed in several areas of preventive medicine [5–11]. In a previous RCT in India, we demonstrated that the delivery of a package of customised, tailored SMS messages based on the transtheoretical model (TTM) of behaviour change was effective compared with standard care, reducing the incidence of type 2 diabetes by 36% over 2 years [9]. In that RCT we recruited working Asian Indian men with persistent prediabetes defined as impaired glucose tolerance on two OGTTs. This method for defining prediabetes (and for assessing progression to diabetes) is time consuming for participants and the healthcare system and is difficult to scale up at societal level.

In the current study, we wished to test the generalisability of the results from the previous trial in India [9]. To do this, first, we included women as well as men; second, as the previous study was relatively small (537 participants), a larger number of participants was recruited; third, we tested the intervention in two ethnically and culturally different environments, India and the UK, using similar primary and secondary outcomes in both countries, with only minor differences reflecting the different populations and settings; finally, we used a more pragmatic method than glucose estimations to define hyperglycaemia, HbA_1c_, as recommended by the WHO [12]. The protocol permitted a comparative pooled analysis of outcomes from the two populations including an exploration of reasons for potential heterogeneity in the results.

## Methods

### Study design and participants

The detailed protocol has been reported previously [13]. In brief, a randomised, controlled clinical trial was conducted over 2 years in people with prediabetes defined by an HbA_1c_ level of ≥42 and ≤47 mmol/mol (≥6.0% and ≤6.4%) (the high prediabetes range). Screening for possible participants took place in workplaces in India and at National Health Service (NHS) Health Checks and in primary care centres in the UK. All participants received structured education for prediabetes and the intervention group received, in addition, SMS messages about lifestyle 2–3 times weekly during the trial. Participants were monitored at baseline and at 6, 12 and 24 months, undertaking repeat assessment of HbA_1c_ and blood glucose levels and completing questionnaires (the Euro quality of life 5 dimension 3 level [EQ-5D-3L], a recent physical activity questionnaire [RPAQ], a TTM of behavioural change questionnaire, and food frequency [UK] or 24 h dietary recall [India]). Physical activity (by accelerometer; ActiGraph GT3X+, ActiGraph, Pensacola, FL, USA) and acceptability of the SMS were monitored at baseline and during follow-up. The primary outcome was progression to diabetes. The secondary outcomes included anthropometric measurements, other cardiovascular risk factors and measures of lifestyle behaviours.

**Fig. 1:**
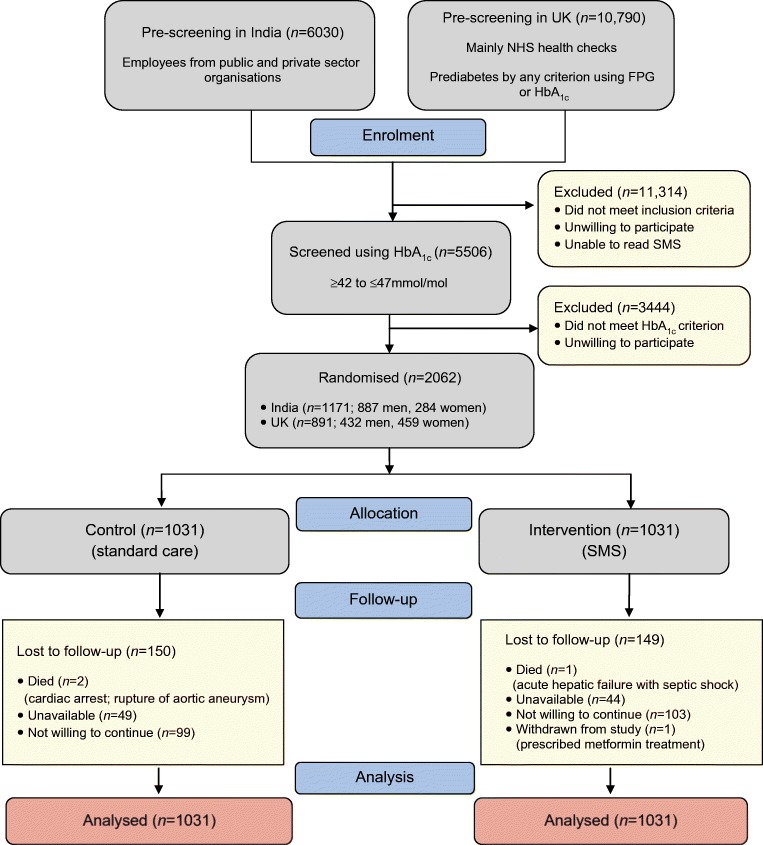
CONSORT diagram of trial profile. FPG, fasting plasma glucose

Figure 1 shows the flow diagram of pre-screening, screening, enrolment and randomisation, and the numbers of participants in the two countries. The total number of participants included in the analysis was 2062 (1031 in the control group, 1031 in the intervention group).

### Pre-screening and screening

In India, pre-screening to identify people at high risk of developing diabetes was undertaken between April of 2012 and November of 2015 in Asian Indian men and women aged 35–55 years in Chennai and surrounding areas. The target population was employees from public and private sector organisations. Following a diabetes awareness programme, participants with no personal history of diabetes or other major physical or mental illness having three or more risk factors, including age 35–55 years, BMI ≥23 kg/m^2^, waist circumference ≥90 cm in men and ≥80 cm in women, first degree family history of type 2 diabetes, history of hypertension or prediabetes, or habitual sedentary behaviour, were selected for further screening using HbA_1c_ [13]. Those with values in the high prediabetes range [14, 15] (≥42 and ≤47 mmol/mol [≥6.0% and ≤6.4%]) were invited to participate in the trial.

In the UK, pre-screening was conducted mainly using the NHS Health Checks programme, which is a cardiovascular and diabetes risk assessment offered routinely and free of charge to people aged 40–74 years without pre-existing diabetes, cardiovascular disease or kidney disease. The programme operates in primary care; people who met the HbA_1c_ entry criteria (≥42 and ≤47 mmol/mol [≥6.0% and ≤6.4%]) were invited to participate in the trial if they fulfilled the other entry criteria. In some primary care centres, screening schemes other than the NHS Health Checks programme were used. Written, informed consent was obtained from all participants.

### Randomisation and masking

Randomisation was performed in India using a computer-generated sequence, to either individually tailored mobile phone SMS messages supplementing baseline lifestyle advice (intervention group) or to a control group that received only the lifestyle modification advice at baseline (1:1). Randomisation in the UK was performed by a commercial organisation in random permuted blocks stratified by sex, age and BMI. Written, informed consent was obtained from the participants and in India permissions had also been obtained from the employers. In both countries, laboratory personnel and investigators were blinded to the participants’ group allocation until the end of the study. Staff involved in delivering the intervention and the participants themselves were, by necessity, not masked.

The study was registered on www.ClinicalTrials.gov (India, NCT01570946; UK, NCT01795833). The trial was registered separately in the two countries since the funding was received from two different national agencies.

### Procedures for text messages

At baseline, in both countries, all trial participants received personalised education and motivation about healthy diet and the benefits of enhanced physical activity. In addition, the intervention group received regular SMS messages, typically 2–3 per week, to provide additional education and motivation. The content of the messages provided by SMS was similar in both countries. The messages used in the previous study in India [9] were modified and expanded. In the UK, a Patient and Public Involvement Group in the National Institute for Health Research Clinical Research Network provided input into the SMS message design and content. The messages provided tips, suggestions and positive reinforcement for healthy behaviours including goal setting, physical activity, dietary planning and personal strategies for lifestyle change. The message content was based on the TTM of behavioural change [16], a stage-based concept categorised by: precontemplation (not ready), contemplation (getting ready), preparation (ready), action and maintenance. We prepared an SMS database and grouped the messages to be appropriate for each TTM stage.

There were 75–80 messages in each TTM stage. Messages were sent to the participants based on the TTM staging performed at each follow-up. The type and content of the messages were changed frequently to avoid repetition. Messages were delivered by commercial service providers. In India, messages were in English and in two local languages and were sent between 06:30 hours and 08:30 hours or after 18:00 hours, as preferred by the participants. In the UK, messages were sent at 10:00 hours on alternate days. The preferred time was ascertained during the follow-up visits so that the messages did not go unnoticed.

Acceptability of SMS in the intervention group was assessed using a short questionnaire [9]. Responses to questions about message content, frequency, ease of understanding, whether messages were considered a disturbance and whether they were perceived as helpful in improving lifestyle were scored as 0 or 1. A total score of 6 was the most acceptable and 0 the least. A modified acceptability questionnaire was used in the UK.

### Lifestyle and quality of life

#### Diet

At baseline, individualised dietary recommendations were delivered to balance food intake and physical activity, and to aim for an appropriate body weight. Advice included: avoidance of simple sugars and refined carbohydrates, reduction of total fat intake (<20 g/day) and inclusion of increased fibre-rich food (e.g. whole grains, legumes, vegetables and fruits). Evaluation was performed using 24 h dietary recall, a method used previously in India [9]. In the UK arm, a food frequency questionnaire was used for calculation of dietary energy intake and major food constituents [17], a method previously validated against 24 h dietary recall [18].

#### Physical activity

Participants who reported being sedentary or who undertook only light physical activity at baseline were advised to walk briskly every day for a minimum of 30 min. People who reported strenuous occupations or sufficient physical activity per day were advised to continue these activities. Physical activity was assessed by self-report using the RPAQ, which has previously been shown to provide a valid estimate of physical activity energy expenditure (PAEE), measured by the gold standard criterion method of doubly labelled water and time spent in different intensity levels [19]. We also assessed physical activity objectively using triaxial accelerometry (ActiGraph GT3X+) which has also been validated against criterion methods [20, 21].

#### Quality of life

The EQ-5D-3L version for India was administered to capture the individuals’ ‘perceived’ quality of life based on the effects of the health intervention [22].

The questionnaire consists of five dimensions (mobility, self-care, usual activities, pain/discomfort, anxiety/depression) and the responses record three levels of severity (no problems/some or moderate problems/extreme problems) within each dimension. The EQ-5D-3L summary measure was calculated using a value set derived from a UK sample since there are no published value sets in Indian populations.

### Biochemical assessments

During the baseline and at each review, anthropometry, blood pressure (mean of two readings using sphygmomanometer), HbA_1c_ and serum lipid profile (total cholesterol, low density lipoprotein, HDL-cholesterol and triacylglycerols) were measured using standard enzymatic procedures with quality control.

### Ethics approvals

In India, the Ethics Review Committee of the India Diabetes Research Foundation and Dr. A. Ramachandran’s Diabetes Hospitals reviewed and approved the study protocol. An independent safety committee assessed study progress with unmasked data at 6 month intervals. In the UK, approval was from the Westminster Research Ethics Committee and site specific assessment (SSA) plus research and development (R&D) approvals were in place at each participating NHS Trust. Imperial College Academic Health Science Centre acted as the main sponsor. Delegated responsibilities were assigned to the participating NHS trusts.

### Outcomes

In the UK, the primary outcome was progression to diabetes as defined by international criteria for fasting plasma glucose or HbA_1c_ at any study review visit or in any healthcare setting. In India, information from study follow-up visits was available; thus, diabetes was defined on HbA_1c_ alone.

The secondary outcomes were body weight and BMI, waist circumference, blood pressure, fasting plasma glucose, lipid levels, proportion achieving HbA_1c_ ≤42 mmol/mol (≤6.0%), acceptability of SMS, dietary variables, physical activity and quality of life.

### Statistical analyses

Based on results from the Indian Diabetes Prevention study [9], a 2 year risk of diabetes in the control group of 25% was assumed. With 2268 participants (1134 per group), the trial had 80% power to detect a relative reduction in risk of 20% as significant at the 5% level, allowing for approximately 4% withdrawals.

Baseline characteristics were summarised by randomised group using mean and standard deviation (continuous variables), median and interquartile range (continuous variables with a skewed distribution), or frequency and percentage (categorical variables).

The primary outcome was compared between intervention and control groups using a discrete-time proportional hazards model with a complementary log-log link function, since the data were interval censored [23], adjusted for country. The multiplicative interactions between randomised group and (1) country and (2) sex were tested using a Wald test. The HR for type 2 diabetes and 95% confidence intervals were reported for the overall trial population, and separately by country (UK/India) and sex, which were the only pre-specified subgroups.

Secondary outcomes, measured at specified time points during follow-up, were analysed using linear regression with random intercepts at the individual level to allow for repeated measures, including the baseline value of the outcome, country, randomised group and time, to estimate an overall intervention effect, and then also using randomised group × time interaction, to estimate intervention effects at each follow-up time. Accelerometer wear time was included in the model for objectively measured physical activity outcomes. Outcomes with a skewed distribution were log-transformed prior to analysis.

The trial was analysed on an intention-to-treat basis. The primary outcome was also analysed in a per-protocol population, which excluded individuals in whom the intervention was not successfully delivered. All analyses were pre-specified [13], and performed using Stata version 14.2 (Stata, College Station, TX, USA).

## Results

Recruitment in India took place between 1 April 2012 and 1 November 2015, and in the UK between 1 June 2013 and 1 November 2017. Follow-up was for 2 years in both countries. The numbers assessed at pre-screening and screening are shown in Fig. 1. In the UK the recruitment took place in multiple primary care settings, mainly using the NHS Health Checks programme; routinely obtained data were scrutinised for eligibility and individuals were asked to participate if they fulfilled the entry criteria.

### Primary and secondary outcomes

In total, 2062 participants were randomised (control: 1031; intervention: 1031). Baseline characteristics were similar in the two randomised groups (Table 1). The mean age was 52.0 (SD 10.3) years, and 64.0% of the participants overall were men.

**Table 1:** Baseline characteristics by randomised group

Variable	Combined India and UK		India		UK	
	Control (*n* = 1031)	Intervention (*n* = 1031)	Control (*n* = 587)	Intervention (*n* = 584)	Control (*n* = 444)	Intervention (*n* = 447)
Sex						
Men	661 (64.1%)	658 (63.8%)	445 (75.8%)	442 (75.7%)	216 (48.6%)	216 (48.3%)
Women	370 (35.9%)	373 (36.2%)	142 (24.2%)	142 (24.3%)	228 (51.4%)	231 (51.7%)
Age (years)	52.0 (10.2)	52.1 (10.3)	45.8 (5.4)	45.8 (5.4)	60.2 (9.3)	60.3 (9.4)
Weight (kg)	79.7 (15.6)	79.0 (15.4)	75.3 (10.8)	74.7 (10.7)	85.4 (18.9)	84.5 (18.5)
BMI (kg/m^2^)	28.9 (4.8)	28.7 (4.7)	27.9 (3.6)	27.5 (3.3)	30.2 (5.8)	30.2 (5.7)
Waist circumference (cm)	97.8 (11.3)	97.5 (11.0)	95.5 (7.5)	95.1 (7.1)	100.8 (14.4)	100.7 (14.0)
Blood pressure (mmHg)						
Systolic	129.3 (16.8)	128.8 (17.0)	127.1 (16.7)	126.8 (17.3)	132.2 (16.4)	131.3 (16.3)
Diastolic	81.5 (10.6)	81.0 (10.6)	82.5 (10.4)	82.1 (10.6)	80.1 (10.8)	79.6 (10.5)
HbA_1c_ (mmol/mol)	43.6 (1.4)	43.6 (1.4)	43.5 (1.4)	43.6 (1.4)	43.8 (1.4)	43.8 (1.4)
HbA_1c_ (%)	6.1 (0.1)	6.1 (0.1)	6.1 (0.1)	6.1 (0.1)	6.2 (0.1)	6.2 (0.1)
Fasting glucose (mmol/l)	5.5 (0.7)	5.5 (0.7)	5.4 (0.7)	5.4 (0.7)	5.6 (0.6)	5.6 (0.6)
Lipid profile (mmol/l)						
Total cholesterol	4.9 (1.1)	4.9 (1.0)	4.7 (0.9)	4.7 (0.8)	5.2 (1.2)	5.2 (1.2)
HDL-cholesterol	1.2 (0.4)	1.2 (0.4)	1.0 (0.2)	1.0 (0.2)	1.5 (0.4)	1.4 (0.4)
LDL-cholesterol	3.0 (0.9)	3.1 (0.9)	3.0 (0.8)	3.0 (0.7)	3.1 (1.1)	3.1 (1.1)
Triacylglycerols	1.3 (1.0–1.8)	1.3 (1.0–1.8)	1.3 (1.0–1.7)	1.3 (1.0–1.7)	1.3 (0.9–1.8)	1.3 (0.9–1.8)

Data are mean (SD) or *n* (%) unless otherwise indicated

^a^Median (interquartile range)

**Fig. 2:**
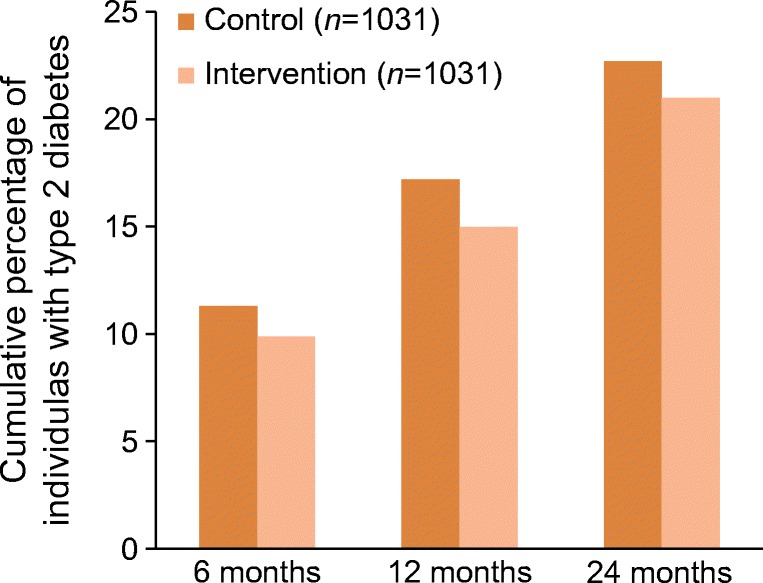
Cumulative percentage of individuals with type 2 diabetes at each follow-up visit

**Fig. 3:**
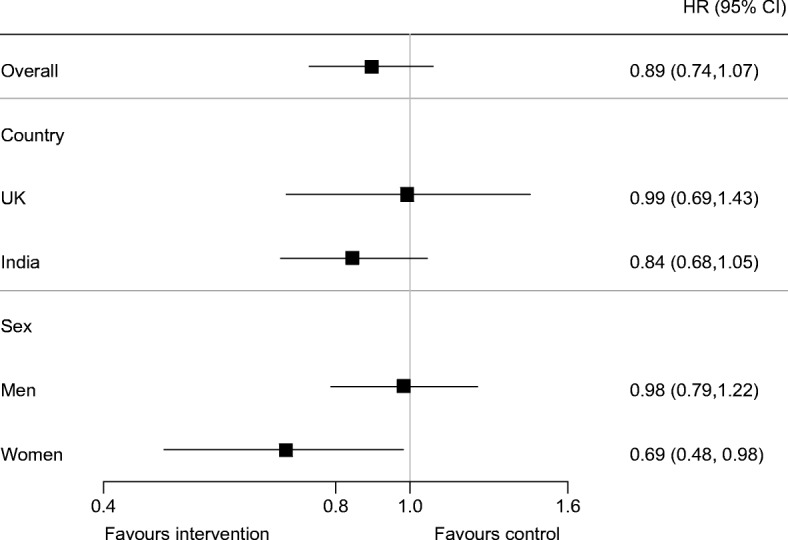
Effect of intervention on primary outcome (the development of type 2 diabetes) overall (*p* = 0.22) and by pre-specified subgroups (intervention × country interaction: *p* = 0.33; intervention × sex interaction: *p* = 0.12)

During the 2 year follow-up period, 234 (22.7%) individuals in the control group and 216 (21.0%) in the intervention group developed diabetes. The cumulative percentage of individuals who developed diabetes at 6, 12 and 24 months in the control and intervention groups is shown in Fig. 2. There was no significant effect of the intervention on the primary outcome (HR for intervention vs control 0.89; 95% CI 0.74, 1.07; *p* = 0.22) (Fig. 3).

**Fig. 4:**
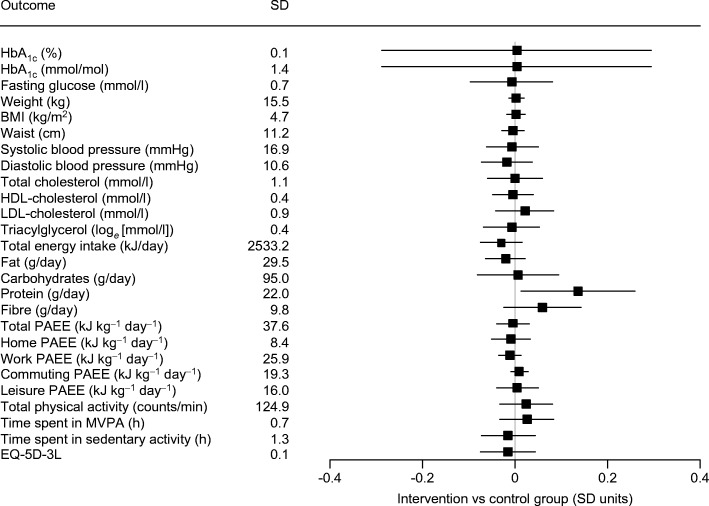
Overall effect of intervention on secondary outcomes (*n* = 2062). Intervention effects represent differences between intervention and control groups, estimated from a linear regression model with random intercepts at the individual level, using measures of the outcome at all follow-up times, and including baseline value of the outcome, country, randomised group and time (months of follow-up). Intervention effects are presented in units of baseline SD of each outcome. Triacylglycerol results are presented using log-transformed values. MVPA, moderate-to-vigorous physical activity

The overall intervention effects on the secondary outcomes are shown in Fig. 4 and in Table 2. Confidence intervals around the estimated effects were wide and overlapped zero. Mean values of most outcomes changed little between baseline and any of the follow-up visits in either randomised group.

**Table 2:** Secondary outcomes at baseline and follow-up

Variable	Control				Intervention			
	Baseline	6 months	12 months	24 months	Baseline	6 months	12 months	24 months
HbA_1c_ (mmol/mol)	43.6 (1.4)	42.9 (5.2)	42.5 (5.8)	43.5 (5.9)	43.6 (1.4)	43.1 (4.8)	42.4 (5.3)	43.7 (5.2)
HbA_1c_ (%)	6.1 (0.1)	6.1 (0.5)	6.0 (0.5)	6.1 (0.5)	6.1 (0.1)	6.1 (0.4)	6.0 (0.5)	6.1 (0.5)
FPG (mmol/l)	5.5 (0.7)		5.6 (0.8)	5.7 (1.0)	5.5 (0.7)		5.7 (0.8)	5.7 (0.8)
Weight (kg)	79.7 (15.6)	79.4 (15.5)	78.6 (15.0)	78.1 (14.9)	79.0 (15.4)	78.5 (15.3)	77.9 (15.1)	77.4 (14.7)
BMI (kg/m^2^)	28.9 (4.8)	28.7 (4.8)	28.5 (4.6)	28.3 (4.6)	28.7 (4.7)	28.5 (4.5)	28.4 (4.6)	28.2 (4.4)
WC (cm)	97.8 (11.3)	97.1 (11.3)	96.7 (10.9)	96.3 (10.8)	97.5 (11.0)	96.7 (10.9)	96.2 (10.7)	96.2 (10.7)
Blood pressure (mmHg)								
Systolic	129.3 (16.8)	128.2 (16.8)	125.1 (15.9)	124.8 (16.8)	128.8 (17.0)	128.4 (17.1)	125.2 (17.0)	124.7 (16.1)
Diastolic	81.5 (10.6)	81.1 (10.4)	80.3 (10.1)	79.6 (9.3)	81.0 (10.6)	80.7 (10.4)	79.9 (10.1)	79.4 (9.1)
Lipid profile (mmol/l)								
Cholesterol	4.9 (1.1)		5.0 (1.1)	5.0 (1.0)	4.9 (1.0)		5.0 (1.0)	5.0 (1.0)
HDL-cholesterol	1.2 (0.4)		1.2 (0.4)	1.2 (0.4)	1.2 (0.4)		1.2 (0.4)	1.2 (0.4)
LDL-cholesterol	3.0 (0.9)		3.0 (0.9)	3.1 (0.9)	3.1 (0.9)		3.1 (0.9)	3.1 (0.9)
Triacylglycerols	1.3 (1.0–1.8)		1.4 (1.0–1.9)	1.3 (1.0–1.9)	1.3 (1.0–1.8)		1.4 (1.0–1.8)	1.3 (1.0–1.8)
Total energy intake (kJ/day)	8303 (2301)	7997 (2060)	7828 (2015)	7654 (1866)	8365 (2746)	7875 (1998)	7871 (1937)	7695 (1894)
Fat intake (g/day)	62.9 (26.4)	59.6 (19.1)	57.9 (19.7)	57.4 (17.4)	63.2 (32.3)	58.2 (19.3)	58.9 (20.9)	57.8 (21.1)
Carbohydrates (g/day)	279.5 (90.5)	270.9 (90.5)	266.8 (87.2)	259.0 (83.8)	282.2 (99.3)	265.7 (87.8)	265.0 (81.9)	257.8 (75.9)
Protein (g/day)	73.6 (20.2)	72.1 (18.9)	70.0 (16.9)	68.8 (17.1)	74.9 (23.6)	72.4 (22.8)	71.9 (20.1)	70.8 (26.1)
Fibre (g/day)	24.8 (9.7)	25.3 (10.3)	25.1 (9.7)	25.0 (9.7)	25.0 (10.0)	25.0 (10.6)	25.4 (9.5)	25.8 (9.4)
PAEE (kJ kg^−1^ day^−1^), RPAQ								
Total	48.3 (39.0)	45.6 (38.0)	48.6 (36.9)	47.9 (35.7)	46.5 (36.2)	44.2 (35.3)	47.5 (35.3)	46.1 (34.6)
Home	2.9 (7.7)	2.5 (6.0)	2.4 (6.1)	2.7 (8.4)	3.1 (9.1)	2.5 (6.6)	2.7 (6.7)	2.3 (5.4)
Work	31.3 (26.2)	31.6 (26.5)	31.8 (26.6)	31.3 (26.0)	32.2 (25.6)	32.1 (26.0)	32.8 (26.4)	32.3 (26.1)
Commuting	11.7 (20.7)	11.3 (20.3)	12.1 (21.0)	12.1 (21.1)	9.4 (17.6)	9.4 (17.6)	9.4 (17.6)	9.7 (18.0)
Leisure	11.7 (17.1)	12.0 (15.3)	11.7 (13.4)	10.7 (11.9)	11.6 (14.9)	12.0 (13.2)	11.8 (13.2)	10.9 (11.7)
Physical activity, ActiGraph								
Total physical activity (counts/min)	412.2 (122.5)	403.7 (126.8)	433.8 (132.0)	400.9 (125.5)	409.0 (127.6)	405.5 (129.5)	439.1 (136.4)	396.5 (124.7)
Time spent in MVPA (h)	1.7 (0.7)	1.7 (0.6)	1.8 (0.7)	1.7 (0.7)	1.7 (0.7)	1.7 (0.7)	1.8 (0.7)	1.6 (0.7)
Time spent in sedentary activity (h)	18.6 (1.3)	18.8 (1.3)	18.3 (1.4)	18.8 (1.3)	18.7 (1.3)	18.7 (1.3)	18.3 (1.4)	18.8 (1.3)
EQ-5D-3L summary measure	0.95 (0.10)	0.94 (0.10)	0.94 (0.10)	0.93 (0.11)	0.94 (0.11)	0.94 (0.10)	0.93 (0.11)	0.93 (0.11)
Body fat (%)^a^	31.2 (5.7)	30.8 (5.7)	31.1 (5.7)	30.9 (5.8)	31.0 (5.3)	31.0 (5.4)	31.2 (5.3)	30.9 (5.5)
HOMA-IR^a^	2.8 (1.9–4.0)		3.2 (2.2–4.6)	3.0 (2.1–4.4)	2.8 (1.8–4.2)		3.2 (2.2–4.5)	3.2 (2.1–4.4)
HOMA-B^a^	138.9 (90.0–206.5)		131.3 (86.6–194.3)	123.5 (86.0–165.6)	135.9 (85.0–199.9)		124.7 (84.6–191.0)	127.7 (80.9–182.9)

Data are mean (SD) or median (interquartile range)

^a^Only measured in India (*n* = 1171)

FPG, fasting plasma glucose; WC, waist circumference; MVPA, moderate-to-vigorous physical activity

For all of the secondary outcomes reported in Table 2, a maximum of 1.3% of individuals had missing values at baseline, except for work PAEE (22.6%), commuting PAEE (22.5%), each of the three ActiGraph physical activity measures (12.6%) and the EQ-5D-3L summary measure (41.1%). The percentages of individuals with missing values were similar in the two randomised groups. When estimating intervention effects using random intercepts linear regression, available data from all time points (including baseline) were included in the model. This assumes that any missing values at either baseline or another time point were missing at random.

Within this trial, the correlation between total physical activity (ActiGraph, counts/min) and total PAEE (kJ kg^−1^ day^−1^, RPAQ) was 0.28 at baseline and 0.32 at 24 months.

Although body weight did not change significantly, there were reductions in estimated intakes of total energy, fat, carbohydrates and protein, and an increase in estimated fibre intake, as assessed by a self-report questionnaire in each group, and as shown in Table 2.

The SMS acceptability questionnaire in India, where the median score out of 6 was 3, showed that messages were generally acceptable. Fewer than 5% of the participants reported that receiving the messages was a disturbance. In the UK the acceptability of SMS varied between 85% at 6 months to 82% at 24 months.

Over the 2 years of follow-up the observed percentages developing diabetes in both intervention and control groups were higher in India (control: 30.3%; intervention: 26.0%) than in the UK (control: 12.6%; intervention: 14.3%). There was no clear evidence of differential effects of the intervention by country or sex (tests of multiplicative interaction: randomised group × country: *p* = 0.33; randomised group × sex: *p* = 0.12) (Fig. 3).

### Analysis of results per protocol in the intervention and control arms

In the per-protocol analysis, the overall results were similar to the intention-to-treat analysis for the primary outcome (HR for intervention vs control 0.95; 95% CI 0.79, 1.16; *p* = 0.63) and for the secondary outcomes.

## Discussion

Although mobile technology is being widely applied in clinical management of a variety of long-term chronic disorders [6, 7, 10, 11, 24, 25] and also in modifying behaviour patterns such as smoking [26], the number of randomised intermediate and long-term studies in prevention of diabetes is limited. In this 2 year RCT involving 2062 participants with prediabetes recruited in two countries, India and the UK, with different ethnic and socio-cultural backgrounds, we have shown that delivery of a behavioural intervention by mobile technology is feasible. However, there was a non-significant risk reduction in the rate of progression of diabetes in 2 years by lifestyle modification using SMS messages.

A previous pilot trial of this intervention that we completed in India alone did find evidence of an effect. However, there are a number of differences between these trials that may explain the inconsistency in findings. First, the former study [9] was conducted in male employees of major industries, whereas the current study included men and women recruited from the general population. It is unlikely that it is the inclusion of women, rather than men alone, that has led to this inconsistency, since a pre-defined subgroup analysis provided weak evidence that the intervention may yield benefit in women. In other diabetes prevention studies, a major sex effect has not been observed [27]. It is more likely that the difference we observed is explained by the selected socio-cultural make-up of the population in the original study compared with the more population-based approach in the current study.

Second, the former study was conducted at a time when SMS messaging was novel in India and it is possible that this novelty and the fact that the messages appeared to come from a healthcare organisation may have influenced their effect. More recently, SMS messaging in India has proliferated, not only for the purpose of personal messaging, but also for mass advertising. Thus, it is possible that in the more recent study the ‘apparently personal’ targeted messaging that was previously effective is now just part of a slew of such messages, and thus may not only be diluted in effect but could also, by being of a similar nature to advertising, be considered an irritant.

Third, the way in which a high-risk prediabetes group was identified was fundamentally different. In the previous study we used the OGTT to define prediabetes and progression to diabetes. This test, and in particular the 2 h glucose, is highly variable and responsive to behaviour change [28], thus making it an excellent way to identify those at risk and the response to intervention. However, we sought not only to test the effectiveness of the intervention, but to do that in a way that was scalable. Unfortunately, the practicalities of the OGTT make it impossible to scale up to a mass intervention. Thus, we utilised the much more practical HbA_1c_ test which could theoretically be employed in a real-life intervention programme to classify prediabetes and progression to diabetes. Although HbA_1c_ has similar biological significance to other measures of glycaemia, for example, in terms of prediction of cardiovascular events [29], a possible disadvantage may be that, as an integrated measure of glucose control over a period of time, HbA_1c_ is less sensitive to behaviour risk factor change, which may partially explain the lower estimate of effect size in this study. Finally, this study, unlike the former study, was conducted both in the UK and in India. It is possible that there could be country differences in the response to such an intervention, for socio-cultural or other reasons. The UK participants were recruited from primary care centres and thus, by definition, were in contact with an organised system of healthcare and would potentially have greater awareness of the importance of healthy lifestyle behaviours. By contrast, the participants in India are likely to have had less access to primary care and thus potentially a lower pre-existing awareness of health-promoting behaviours and a greater potential to benefit from this form of individual-level targeted prevention strategy.

Overall, the observed progression rate from prediabetes to diabetes was greater in India than in the UK. The rate in India is compatible with that in our previous Indian study [9] and the approximately 50% lower progression rate in the UK is consistent with other recent UK studies [30]. Previous analysis of the Diabetes Prevention Program (DPP) intervention within a single country has shown no differences in the risk of progression to diabetes from prediabetes between ethnic groups [31]. Our study shows that the rate of progression is markedly different between countries. We were not able in a pre-specified analysis to demonstrate significant differences in any intervention effect between the two countries, but this analysis may be under-powered to investigate differences in a low effect size. The low incidence of diabetes in the UK arm of this trial may have limited our ability to detect an effect of the intervention. Nor were we able to demonstrate significant differences in the secondary endpoints. Some of the apparent improvements that were observed in self-reported dietary components may be explained by reporting bias.

The results of this trial need to be set in the context of results of intervention evaluations elsewhere. In a recent trial in Denver, USA, Fischer et al evaluated text messaging as an aid to achieving weight loss in individuals with prediabetes [32]. Over 12 months, a clinically significant benefit in terms of weight loss was observed in the intervention group, but at 1 year HbA_1c_ levels did not differ between the groups. A similar impact on weight was observed in a 12 month trial of a low-intensity lifestyle programme in Australian women [33], but although this intervention included monthly text messages on healthy behaviour, these messages were delivered in addition to phone coaching and provision of a programme manual, making it difficult to isolate the effect of the messages alone.

The utility of SMS in improving adherence to antiretroviral therapy [34] and smoking cessation has also been reported [35].

In a recent study in Bangladesh, a community-based intervention with facilitator-led group meetings was effective in preventing type 2 diabetes when an SMS-based intervention alone was not [36]. These studies and our own are compatible with the conclusions from a recent systematic review of electronically delivered weight loss programmes [37] that electronic delivery of lifestyle advice and motivation alone may be less effective than when supplemented with remote counselling or counselling in person. Cultural differences may also influence outcome and variability in results [24], although no major differences were observed in our study between effects in India and the UK.

Future studies should be powered to detect small intervention effects which may not be meaningful at an individual level, but which might be meaningful if scaled across a population level. We would also suggest that studies should be established to investigate more thoroughly the contextual factors that may influence the effectiveness of this type of intervention.

## Data Availability

The data that support the findings of this study are available from the corresponding author on request.

## Abbreviations

EQ-5D-3L: Euro quality of life 5 dimension 3 level
NHS: National Health Service
PAEE: Physical activity energy expenditure
RPAQ: Recent physical activity questionnaire
SMS: Short message service
TTM: Transtheoretical model
